# Optimizing black cumin (*Nigella sativa* L.) productivity under semi-arid conditions: effects of sowing date and seed rate on seed yield and oil content

**DOI:** 10.7717/peerj.21652

**Published:** 2026-08-17

**Authors:** İsmail Demir

**Affiliations:** Faculty of Agriculture, Field Crops Department, Kırşehir Ahi Evran University, Kırşehir, Türkiye

**Keywords:** *Nigella sativa*, Sowing date, Seed rate, Seed yield, Oil content, Semi-arid conditions, Heat stress

## Abstract

Black cumin (*Nigella sativa* L.) is a very important medicinal plant; however, its productivity in rainfed semi-arid environments is increasingly threatened by changing climate conditions. Mitigating the negative impacts of climate change may be possible through agricultural practices such as early sowing and seed rate. This study was conducted to determine the effects of different sowing dates and seed rates on the yield and yield parameters of black cumin over two consecutive growing seasons (2021–2022) under rainfed semi-arid climate conditions. The experiment was carried out using a split plot design with five sowing dates (early March to late April) and three seed rates (10, 20, and 30 kg ha^−1^). Results indicated that sowing date was the primary determinant of crop performance. Early sowing (early to mid-March) significantly extended both vegetative and reproductive phases, resulting in improved plant growth, yield components, and seed filling. This shift in crop development reduced exposure to terminal heat stress and led to the highest seed yield (1,099.3 kg h^−1^) and oil productivity. Multiple regression analysis revealed that mean temperature during the reproductive phase was the dominant climatic factor limiting yield (*P* < 0.001), while precipitation had a positive but less pronounced effect (*P* < 0.01). In addition, seed rate significantly affected yield, with lower seed rate (10 kg ha^−1^) enhancing both seed yield and oil content by reducing intra specific competition. Delayed sowing shortened the growth cycle and reduced yield by approximately 65%. Despite interannual climatic variability, consistent responses across years indicate that early sowing combined with lower seed rates is an effective strategy to improve productivity under semi-arid conditions. These findings demonstrate that optimizing sowing date and seed rate not only enhances yield and oil content but also provides a practical adaptation strategy to mitigate the adverse effects of climatic stress.

## Introduction

Black cumin (*Nigella sativa* L.), a prominent member of the Ranunculaceae family, is a strategic medicinal plant characterized by an increasing global demand in both the pharmaceutical and food industries (Şen, Kar & Tekeli, 2010; Yimer et al., 2019). Its seeds are highly valued for their rich fixed and volatile oil components, particularly thymoquinone. However, the biosynthesis of these secondary metabolites is heavily dependent on genotype × environment (G × E) interactions (Villalobos, Sadras & Fereres, 2017). While the potential for secondary metabolite production is genetically determined, environmental factors and cultural practices are suggested to play a decisive role in realizing this potential and determining final yield and quality traits (Kara, Katar & Baydar, 2015).

Currently global climate change characterized by rising temperatures and erratic precipitation regimes poses a significant threat to agricultural sustainability particularly in semi-arid regions (Kayacetin, 2022; Pörtner et al., 2022). For crops with sensitive phenological stages like N. sativa exposure to water scarcity and high temperatures during the reproductive phase is defined as terminal heat stress. This stress is evaluated as a factor that restricts photosynthetic activity and shortens the seed filling duration which consequently leads to substantial yield losses (Ghiyasi et al., 2019). In this context synchronizing the phenological development of the crop with optimal environmental windows has become a critical necessity for sustainable production rather than a mere agronomic preference.

Sowing date is considered the most fundamental agronomic decision, as it determines the photoperiod, temperature, and moisture regimes the plant will encounter throughout its life cycle (Sadeghi, Rahnavard & Ashrafi, 2009). Optimizing the sowing window is seen to enable the crop to utilize a ”drought escape” mechanism; early sowing prolongs the vegetative growth period, enhances biomass accumulation, and allows the plant to complete its critical flowering and seed set stages before the onset of extreme summer heat (D’Antuono, Moretti & Lovato, 2002). Conversely, delayed sowing practices have been identified as a factor that forces plants into premature senescence (forced maturity), correlating with significant reductions in thousand seed weight and oil content.

However, optimizing the sowing date alone is insufficient; seed rate must also be regulated to ensure the efficient use of limited resources such as water, light, and nutrients. An optimized seed rate is hypothesized to establish a delicate balance between individual plant performance and population level yield. It is understood that while low seed rates fail to maximize yield per unit area due to inadequate plant populations, excessively high seed rates exacerbate intraspecific competition, thereby suppressing branching, capsule formation, and secondary metabolite synthesis (Toncer & Kizil, 2004).

While the individual effects of sowing date and seed rate on black cumin have been documented in various studies, the synergistic impact of these factors on the crop’s phenological plasticity remains a significant knowledge gap especially under thermal stress in semi-arid transition zones. This study was designed to address this gap by evaluating the effectiveness of adjusted sowing schedules as a phenological escape strategy against climate induced stressors. Furthermore, the research aims to identify an optimized seed rate that maximizes both seed and fixed oil yields to provide a robust and resilient management framework for rainfed agricultural systems.

## Materials & Methods

### Experimental site and climatic conditions

Field experiments were conducted during the 2021 and 2022 growing seasons at the Kırşehir Ahi Evran University Research and Application Farm in Türkiye (39°11′N, 34°10′E; 1,020 m elevation). The region has a semi-arid climate characterized by cold winters and hot dry summers where crop production depends heavily on seasonal rainfall.

Prior to the experiment representative soil samples were collected from 0–30 cm and 30–60 cm depths for physical and chemical analysis at the Kırşehir Ahi Evran University Central Laboratory (Table 1). The soil was classified as clay loam with a slightly alkaline pH (7.59 7.63). It was non saline and poor in organic matter (<1.81%) while being low in available phosphorus but sufficient in potassium (Kacar, 1994). These soil characteristics are typical of the Central Anatolian steppe and represent the nutrient limitations frequently encountered in rainfed agriculture.

**Table 1:** Physical and chemical properties of the soil at the experimental site.

Depth cm	Saturation (%)	pH	EC (mmhos cm^−1^)	CaCO_3_ (%)	P_2_O_5_ (kg ha^−1^)	Organic matter (%)	K_2_O (kg ha^−1^)	Total salt (%)
0–30	55	7.59	0.52	27.9	21.4	1.81	666.2	0.02
30–60	55	7.63	0.56	28.39	22.9	1.64	514.7	0.02

Climatic data for the experimental years and long-term averages (1970–2022) are presented in Table 2. Total precipitation during the growing season (March–August) was 165.4 mm in 2021 and 153.2 mm in 2022, both lower than the long-term average of 175.9 mm. Rainfall distribution also differed between years, with relatively greater precipitation during the vegetative stages in 2021. Maximum temperatures increased progressively throughout the growing season and reached 39.0 °C in July 2021 and 36.9 °C in July 2022, exceeding the corresponding long-term averages. Similarly, August maximum temperatures remained above the long-term mean. These elevated temperatures coincided with the reproductive period of late-sown crops and indicate the occurrence of terminal heat stress. Minimum temperatures generally followed seasonal patterns and were comparable to long-term values. Overall, the climatic conditions during the experimental years provided contrasting thermal and moisture environments that were suitable for evaluating black cumin responses to sowing date and seed rate under rainfed semi-arid conditions.

**Table 2:** Monthly maximum (Tmax), minimum (Tmin), and mean air temperatures (°C), relative humidity (%), and total precipitation (mm) during the 2021 and 2022 growing seasons compared with long-term averages (LT, 1970–2022).

Month	Maximum temperature (°C)			Minimum temperature (°C)			Mean temperature (° C)			Relative humidity (%)			Total precipitation (mm)		
	2021	2022	LT	2021	2022	LT	2021	2022	LT	2021	2022	LT	2021	2022	LT
March	19.8	18.2	20.9	−6.3	−9.1	−7.5	4.5	1.2	5.3	65.5	68.8	66.6	95.2	52.9	40.5
April	29.7	30.0	25.5	−1.8	−4.3	−1.8	12.0	13.9	10.8	56.5	43.0	62.5	19.4	7.1	40.6
May	33.0	32.4	29.0	4.0	3.3	3.1	18.2	14.9	15.5	45.3	59.0	60.1	9.2	56.3	44.3
June	33.1	34.0	32.9	6.1	10.2	7.6	19.3	20.8	19.7	55.2	56.5	54.9	35.1	36.8	34.6
July	39.0	36.9	35.9	11.7	12.5	11.4	24.9	23.7	23.1	40.5	43.8	46.7	0.9	0.1	8.1
August	38.1	37.0	35.6	13.6	16.6	11.4	24.3	27.7	23.1	43.4	37.3	47.3	5.6	0	7.8
Mean/Total	32.1	31.4	30.0	4.6	4.9	4.0	17.2	17.0	16.3	51.1	51.4	56.4	165.4	153.2	175.9

**Notes.**

LT, long-term average (1970–2022).

### Plant material and experimental design

The black cumin population ‘Çameli’, which is widely adapted to the region, was used as the plant material. The study was arranged in a split plot design based on a Randomized Complete Block Design (RCBD) with three replications. The main plots were assigned to five sowing dates (10 March, 20 March, 1 April, 15 April, and 30 April), while the sub plots consisted of three seed rates (10, 20, and 30 kg ha^−1^).

Each sub-plot consisted of four rows, 5 m in length, with 25 cm inter-row spacing, resulting in a plot size of 5 m^2^. Seeds were sown manually at a depth of 1–2 cm. No supplemental irrigation was applied throughout the growing seasons; the crop relied entirely on rainfall (rainfed conditions). Weed control was performed manually as required to keep the plots free of weeds.

### Data collection

Phenological observations, including days to 50% flowering and days to physiological maturity, were recorded on a plot basis. Morphological traits (plant height, number of lateral branches, and number of capsules per plant) and yield components (seeds per capsule) were measured on 10 randomly tagged plants harvested from the inner rows of each plot to avoid border effects. For seed yield determination, plants were harvested from the central area of each plot (2.25 m^2^) at full maturity. The harvested plants were air dried, threshed, and cleaned. Thousand seed weight (TSW) was determined by weighing four replicates of 100 seeds and converting the values to 1000 seed weight. Seed yield was extrapolated to kilograms per hectare (kg ha^−1^).

### Oil extraction and analysis

The seed oil content was determined by automated Soxhlet extraction using a Gerhardt Soxtherm SOX414 (C. Gerhardt GmbH & Co. KG, Königswinter, Germany) apparatus. Fine ground seed samples (five g) were subjected to extraction with n-hexane as the solvent. The automated process included the boiling, rinsing, and solvent recovery stages, maintained at a specific extraction temperature for 6 h. After extraction, the remaining solvent was evaporated, and the oil samples were dried in an oven until a constant weight was achieved. The total oil content was calculated on a dry weight basis and expressed as a percentage (%) of the total seed weight.

### Determination of agrometeorological indices

To quantify the thermal and environmental pressure on black cumin across different sowing windows, the following indices were calculated:

(a) Growing Degree Days (GDD): Calculated from sowing to physiological maturity using the standard equation:

\begin{eqnarray*}GDD=\sum \frac{ \left( {T}_{max}+{T}_{min} \right) }{2} -{T}_{base} \end{eqnarray*}

where T_max_ and T_min_ are daily maximum and minimum temperatures, respectively, and Tbase is the base temperature, established as 5 °C for black cumin according to previous studies (Ghaderi, Soltani & Sadeghipour, 2008; Ghamarnia, Miri & Ghobadei, 2014; Paredes et al., 2025).

(b) Heat Use Efficiency (HUE): Expressed as the amount of seed yield produced per unit of thermal accumulation (Rao, Singh & Singh, 1999):

\begin{eqnarray*}\mathrm{HUE}({\mathrm{kg~ ha}}^{-1}~\textdegree {\mathrm{C~ day}}^{-1})= \frac{ \left( \mathrm{Seed~ Yield} \right) }{ \left( \mathrm{Total~ GDD} \right) } \end{eqnarray*}

(c) Precipitation Use Efficiency (PUE): Calculated to assess water productivity (Williams, Long & Reardon, 2020):

\begin{eqnarray*}PUE \left( {\mathrm{kg~ ha}}^{-1}~{\mathrm{mm~ day}}^{-1} \right) = \frac{ \left( \mathrm{Seed~ Yield} \right) }{ \left( \text{Cumulative Rainfall} \right) .} \end{eqnarray*}

### Statistical analysis

Data obtained from the two growing seasons were subjected to a combined Analysis of Variance (ANOVA) using MSTAT-C statistical software. The homogeneity of error variances was tested prior to the combined analysis. The model included year, sowing date, and seed rate as fixed factors. When the *F*-test indicated significant differences, mean comparisons were performed using the Least Significant Difference (LSD) test at the *P* < 0.05 probability level. Furthermore, Pearson correlation analysis was conducted to determine the relationships between agrometeorological indices (GDD, rainfall) and yield parameters. Simple linear regression models were employed to quantify the response of seed yield and oil content to maximum temperature fluctuations during the reproductive phase. Additionally, to evaluate the integrated impact of climatic factors on seed yield, a multiple linear regression analysis was performed using total rainfall and mean maximum temperature during the reproductive stage as independent variables. This model approach aimed to isolate the specific contribution of terminal heat stress and moisture availability to the final yield components. Regression analyses were conducted using Python (utilizing the pandas, scipy, and statsmodels libraries). Principal component analysis (PCA) was performed using the scikit-learn library after data standardization, and graphical visualizations were generated using the matplotlib library.

## Results

### Agrometeorological dynamics and resource use efficiency

The response of black cumin to different sowing dates was characterized by significant shifts in agrometeorological indices. As sowing was delayed from March 10 to April 30, total Growing Degree Days (GDD) accumulated more rapidly, particularly during the generative phase (Table 3). Seed filling duration was shortened under delayed sowing conditions.

**Table 3:** Agrometeorological indices and resource use efficiency.

**Year**	**Sowing date**	**Yield (kg/ha)**	**Total GDD**	**Total rain (mm)**	**HUE (kg/ha ^∘^C)**	**PUE (kg/ha mm)**
**2021**	10 March	1,052.6	1,815.3	159.6	0.581	6.60
	20 March	1.150.0	1.906.3	136.3	0.604	8.44
	01 April	1,009.6	2,034.8	70.2	0.497	14.38
	15 April	720.0	1,958.8	60.6	0.368	11.88
	30 April	403.2	1,941.9	50.8	0.207	7.94
**2022**	10 March	989.8	1,732.3	109.1	0.572	9.07
	20 March	1,048.5	1,879.8	103.2	0.559	10.16
	01 April	906.6	2,060.3	100.3	0.441	9.04
	15 April	675.9	1,945.1	93.6	0.348	7.22
	30 April	370.7	1,947.8	93.6	0.190	3.96

Heat Use Efficiency (HUE) declined markedly with delayed sowing. In 2021, the maximum HUE was recorded as 0.604 kg ha^−^^1^^∘^C day^−^^1^ for the March 20 sowing, while this value decreased to 0.207 kg ha^−^^1^^∘^C day^−^^1^ for the latest sowing on April 30. A similar trend was observed in 2022, where HUE reached its lowest value of 0.190 kg ha^−^^1^^∘^C day^−^^1^.

Differences in precipitation were observed between years and sowing dates. In 2022, the April 30 sowing received 93.6 mm of rainfall compared to 50.8 mm in 2021. Despite this increased precipitation, seed yield was lower, and Precipitation Use Efficiency (PUE) declined from 7.94 to 3.96 kg ha^−^^1^ mm^−^^1^.

Additionally, although precipitation differed by approximately 16 mm between the earliest and latest sowing dates in 2022, significant differences in seed yield were still observed.

### Phenological traits and growth characteristics

The combined analysis of variance showed that days to flowering were significantly affected (*P* < 0.01) by year, sowing date, and seed rate, whereas days to maturity were significantly influenced (*P* < 0.01) by sowing date and the sowing date × seed rate interaction (Table 4).

**Table 4:** Summary of ANOVA significance levels for the effects of year, seed rate, sowing date, and their interactions on agronomic traits of *Nigella sativa* L.

Trait	CV (%)	Y	SD	SR	Y × SD	Y × SR	SD × SR	Y × SD × SR
DTF	2.04	^**^	^**^	^**^	ns	ns	ns	ns
DTM	1.87	ns	^**^	ns	ns	ns	^**^	ns
PH	6.41	ns	^**^	ns	ns	ns	^**^	ns
LB	13.45	ns	^**^	^**^	ns	ns	ns	ns
CN	12.27	ns	^**^	^**^	ns	ns	ns	ns
SNUM	4.32	ns	^**^	^**^	ns	^**^	^**^	ns
TSW	5.98	ns	^**^	^**^	ns	ns	^*^	ns
SY	6.08	^**^	^**^	^**^	ns	ns	^**^	ns
OC	2.59	ns	^**^	^**^	^**^	ns	^**^	ns
OY	7.13	ns	^**^	^**^	ns	ns	^**^	ns

**Notes.**

* *p* < 0.05.

** *p* < 0.01.

ns not significant DTF Days to Flowering DTM Days to Maturity PH Plant height LB Lateral Branches CN Capsules per Plant SNUM Seeds per Capsule TSW Thousand seed weight SY Seed yield OC Oil Content OY Oil yield CV Coefficient of Variation, Y Year SD Sowing date SR Seed rates

Days to flowering decreased with delayed sowing (Table 5). The longest time to flowering (86.9 days) was recorded at the earliest sowing date (10 March), whereas flowering occurred earlier (63.3 days) at the latest sowing date (30 April). A significant year effect was also observed.

**Table 5:** Effects of year, seed rate, and sowing time on days to flowering, days to maturity, plant height, lateral branches, and capsule number of *Nigella sativa* L. For each main effect, means in rows and columns followed by the same letter are not significant at *P* ≤ 0.05 according to the Least Significant Difference (LSD) test.

Treatments		Days to flowering	Days to maturity	Plant height (cm)	Lateral branches (no. plant^−1^)	Capsules (no. plant^−1^)
Year	2021	76.38 a	134.69	33.52	6.29	10.16
	2022	75.10 b	134.42	33.05	6.03	9.71
LSD (0.05)		1.08	ns	ns	ns	ns
Seed rate	10 kg ha^−1^	74.77 b	134.39	33.23	6.53 a	10.22 a
	20 kg ha^−1^	75.51 b	134.71	33.17	6.36 a	10.43 a
	30 kg ha^−1^	76.94 a	134.56	33.44	5.60 b	9.15 b
LSD (0.05)		0.80	ns	ns	0.43	0.63
Sowing date	10 March	86.89 a	147.39 a	43.72 a	8.04 a	11.11 b
	20 March	81.86 b	143.37 b	40.09 b	8.12 a	13.67 a
	1 April	77.73 c	139.95 c	36.06 c	7.16 b	13.22 a
	15 April	68.91 d	125.29 d	25.15 d	4.53 c	7.43 c
	30 April	63.30 e	116.77 e	21.39 e	2.96 d	4.24 d
LSD (0.05)		1.37	1.55	1.23	0.49	0.79
Mean		75.74	134.55	33.28	6.16	9.93

Seed rate significantly affected days to flowering, with higher seed rates slightly increasing the duration to flowering.

**Figure 1:**
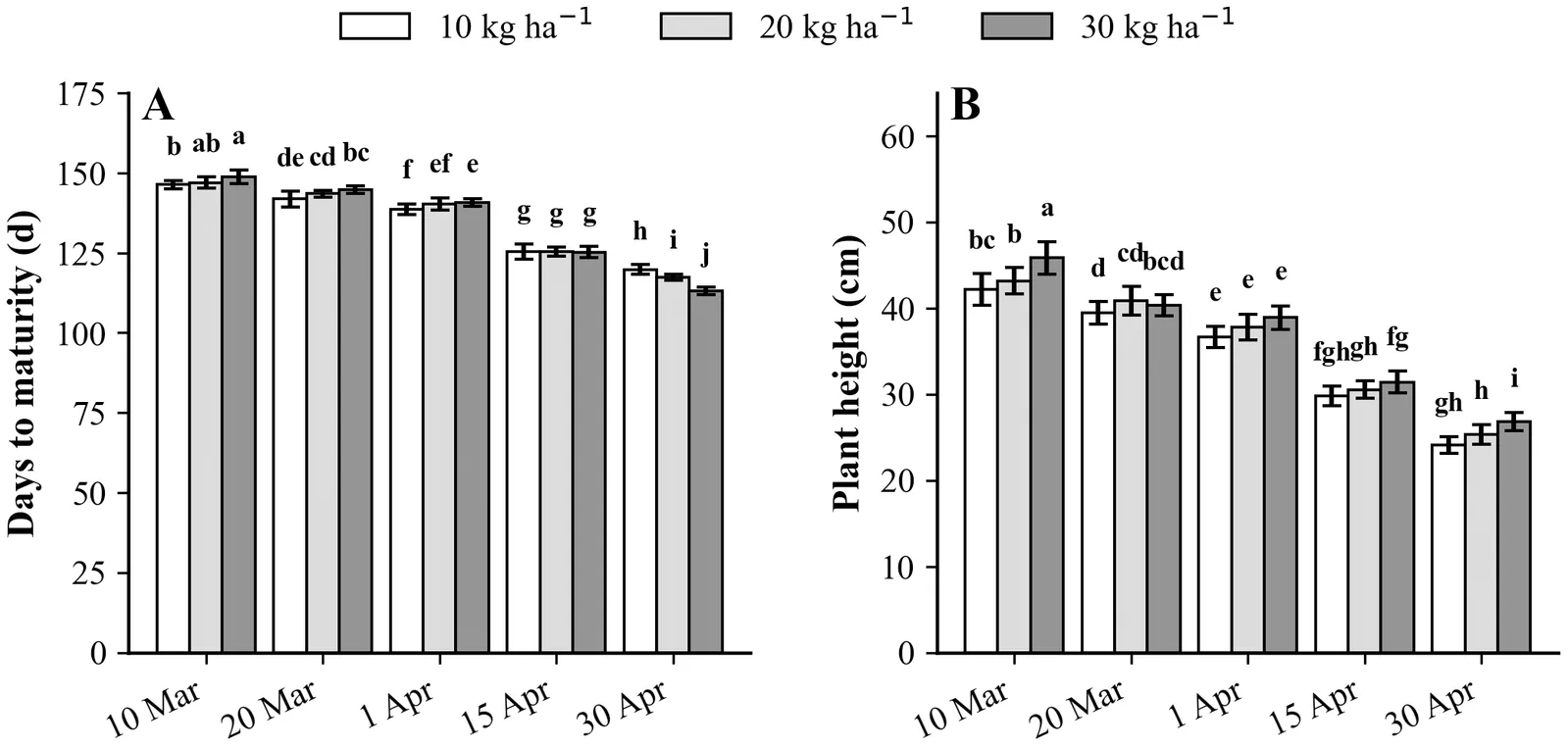
Interactive effects of sowing date (SD) and seed rate (SR) on (A) days to maturity and (B) plant height of black cumin. Bars represent mean values of two growing seasons (2021–2022), and error bars indicate standard deviation. Different letters above the bars indicate statistically significant differences among the SD × SR combinations at *P* ≤ 0.05 according to Fisher’s Least Significant Difference (LSD) test.

Days to maturity were primarily controlled by sowing date, with delayed sowing significantly shortening the crop growth cycle (Table 5). A significant sowing date × seed rate interaction was also observed (Table 4; Fig. 1A), indicating that the effect of seed rate on maturity depended on the sowing date. Despite this interaction, sowing date remained the dominant factor regulating the total crop duration.

Plant height was significantly affected (*P* < 0.01) by sowing date and the sowing date × seed rate interaction, whereas the main effect of seed rate was not significant (Table 4). The highest plant height (43.7 cm) was recorded at the earliest sowing date (10 March), while the lowest (21.4 cm) was observed at the latest sowing date (30 April) (Table 5; Fig. 1B).

The number of lateral branches per plant was significantly influenced (*P* < 0.01) by both sowing date and seed rate. Early sowing produced approximately 8 branches per plant, whereas late sowing reduced this value to 2.96. Higher seed rates slightly reduced the number of branches.

Capsules per plant were significantly affected (*P* < 0.01) by sowing date and seed rate. The highest capsule numbers (up to 13.67 per plant) were observed in March and early April sowings, while the lowest values (4.24 capsules per plant) were recorded at the latest sowing date.

### Yield components and seed yield

**Figure 2:**
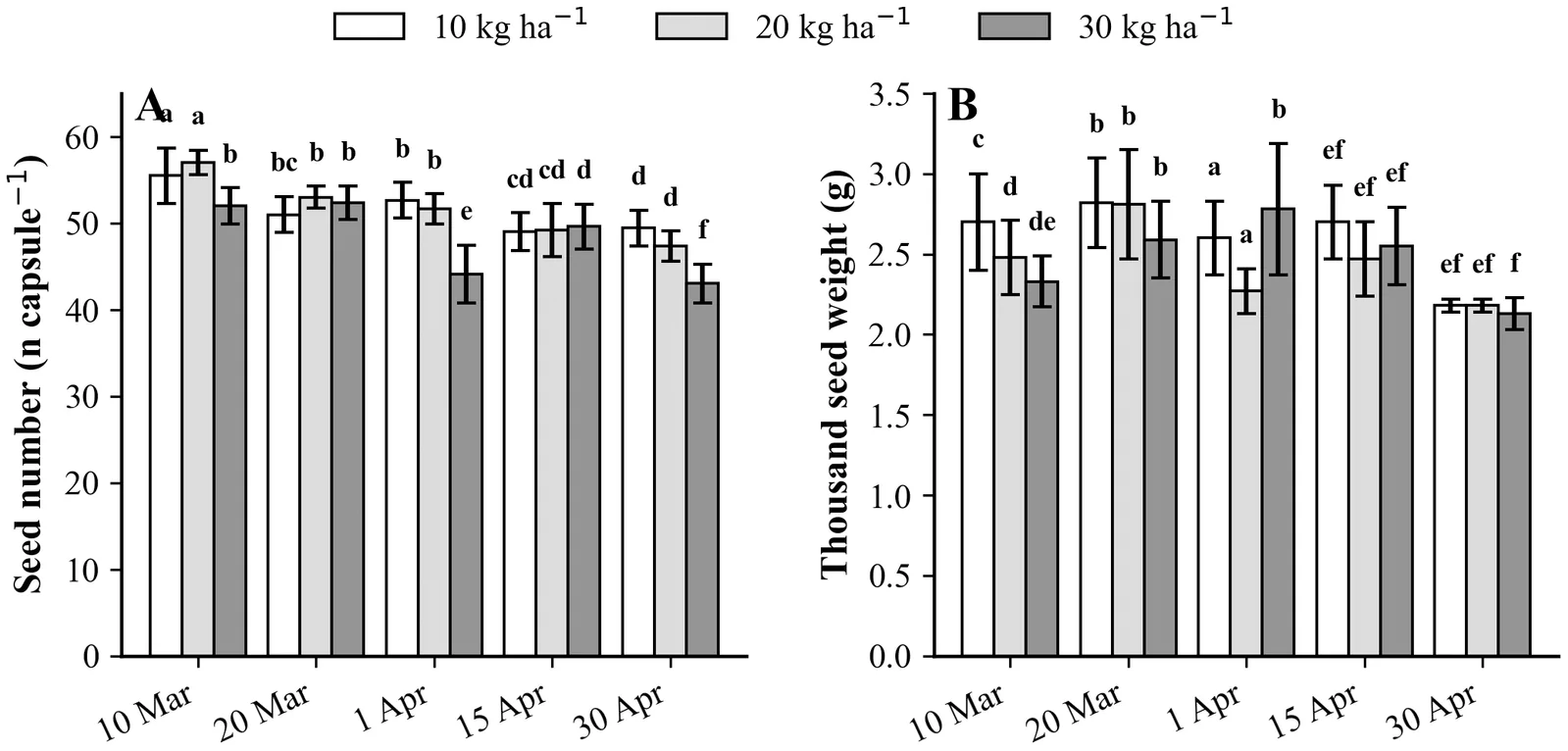
Effects of sowing date (SD) and seed rate (SR) on (A) seed number per capsule and (B) thousand seed weight of black cumin. Values are means of two growing seasons (2021–2022). Error bars indicate standard deviation. Different letters indicate significant differences at *P* < 0.05 (LSD test).

The number of seeds per capsule was significantly affected (*P* < 0.01) by sowing date and seed rate, whereas the main effect of year was not statistically significant (Table 4). March sowings produced the highest seed numbers, while delayed sowing resulted in a marked reduction (Table 6). A significant sowing date × seed rate interaction was observed (Fig. 2A).

**Table 6:** Effects of year, seed rate, and sowing time on seeds per capsule, thousand-seed weight, seed yield, oil content, and oil yield of *Nigella sativa* L. For each main effect, means in rows and columns followed by the same letter are not significant at *P* ≤ 0.05 according to the Least Significant Difference (LSD) test.

Treatments		Seeds per capsule (no.)	1000-seed weight (g)	Seed yield (kg ha^−1^)	Oil content (%)	Oil yield (kg ha^−1^)
Year	2021	50.05	2.74	870.4 a	30.75	276.39
	2022	50.21	2.72	791.0 b	30.44	253.67
LSD (0.05)		ns	ns	67.08	ns	ns
Seed rate	10 kg ha^−1^	51.31 a	2.82 a	890.37 a	30.71	284.55 a
	20 kg ha^−1^	51.65 a	2.75 a	853.37 b	30.64	273.30 b
	30 kg ha^−1^	47.43 b	2.60 b	748.32 c	30.44	237.24 c
LSD (0.05)		1.13	0.09	26.34	ns	10.25
Sowing date	10 March	54.83 a	2.51 c	1,011.2 b	34.78 a	351.71 b
	20 March	52.34 b	3.23 b	1,099.3 a	33.74 b	370.74 a
	1 April	49.49 c	3.50 a	958.1 c	33.24 b	318.78 c
	15 April	48.73 c	2.23 d	698.0 d	27.65 c	192.63 d
	30 April	45.24 d	2.17 d	387.0 e	23.56 d	91.29 e
LSD (0.05)		1.20	0.08	33.14	0.80	13.48
Mean		50.13	2.73	830.7	30.59	265.03

Thousand seed weight (TSW) was significantly influenced by sowing date and seed rate (*P* < 0.01), as well as their interaction (*P* < 0.05), whereas the year effect was not significant (Table 4). Higher TSW values were recorded under early sowing, while delayed sowing reduced seed weight (Table 6). Lower seed rates resulted in higher TSW values. A significant sowing date × seed rate interaction was also observed (Fig. 2B).

Seed yield was significantly affected (*P* < 0.01) by year, sowing date, seed rate, and their interaction (Table 4). The highest yield (1,099.3 kg ha^−1^) was obtained at mid-March sowing, followed by early March (1,011.2 kg ha^−1^), whereas the lowest yield (387.0 kg ha^−1^) was recorded at late April sowing (Table 6).

Seed yield decreased by approximately 65% with delayed sowing. Lower and moderate seed rates (10 and 20 kg ha^−1^) resulted in higher yields compared to the highest seed rate (30 kg ha^−1^). A significant sowing date × seed rate interaction indicated that the effect of plant population varied depending on the sowing window.

### Oil content, oil yield and regression analysis

**Figure 3:**
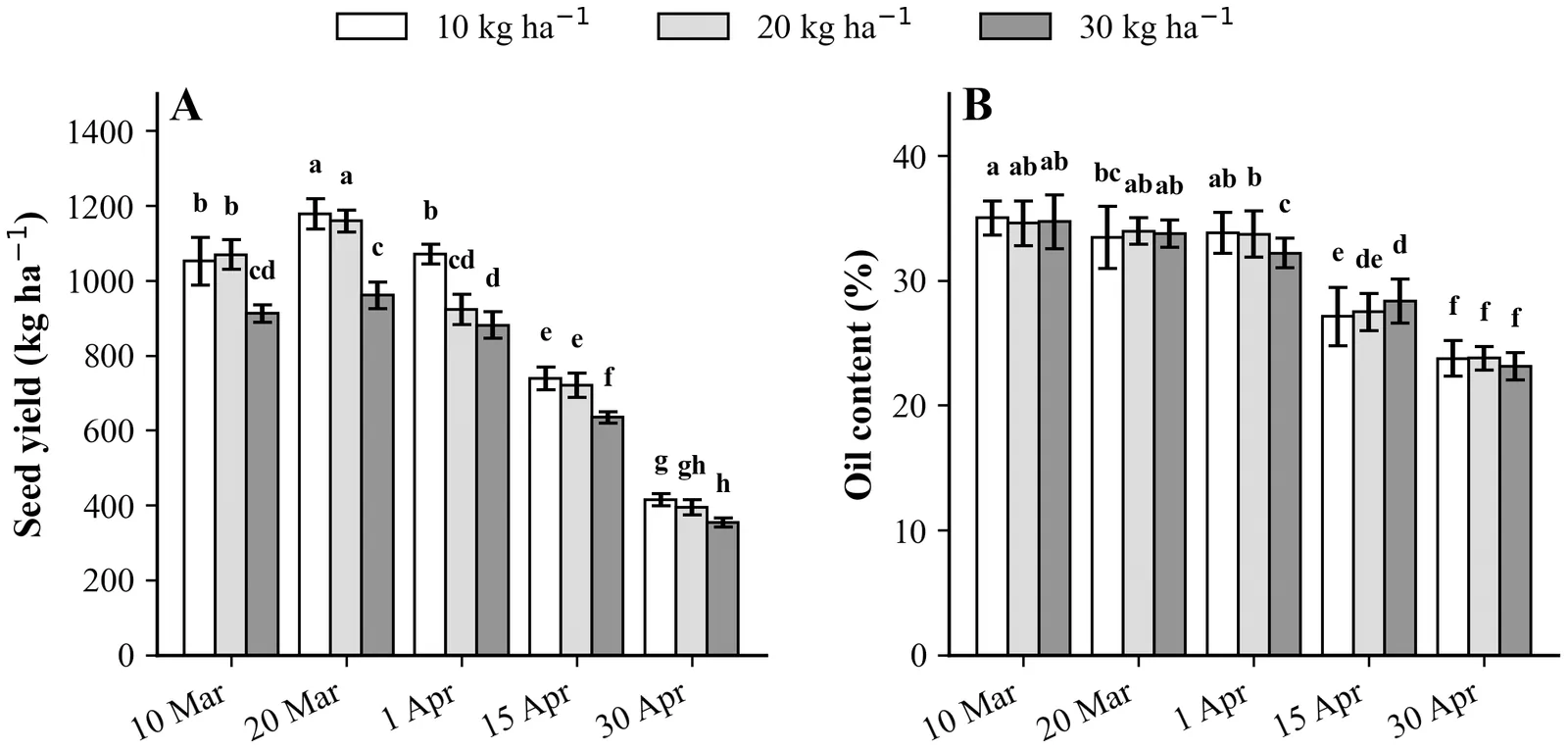
Effects of sowing date (SD) and seed rate (SR) on (A) seed yield and (B) oil content of black cumin. Values are means of two growing seasons (2021–2022). Error bars indicate standard deviation. Different letters indicate significant differences at *P* < 0.05 (LSD test).

Oil content was significantly affected (*P* < 0.01) by sowing date and seed rate, whereas the year effect was not statistically significant (Table 4). Higher oil content was observed under early and optimal sowing dates, while delayed sowing reduced oil concentration (Table 6). Lower seed rates resulted in higher oil content. A significant sowing date × seed rate interaction was also observed (Fig. 3B).

**Figure 4:**
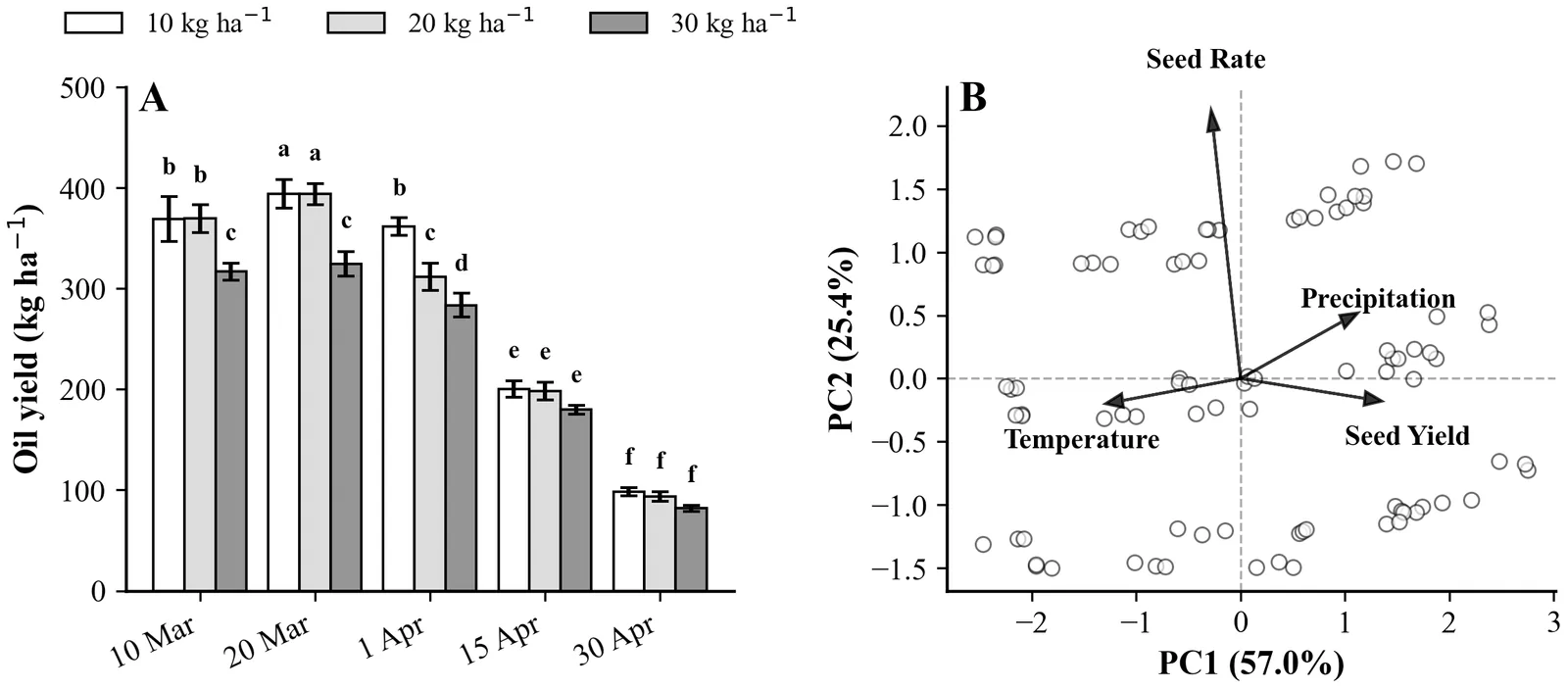
Combined assessment of (A) oil yield and (B) Principal Component Analysis (PCA) biplot showing the relationships between environmental factors and yield parameters. In the bar chart, different letters indicate significant differences at *P* ≤ 0.05. In the PCA biplot, arrows indicate the direction and strength of the influence of environmental variables on yield.

The main effect of year on oil yield was not statistically significant (Table 4). Sowing date significantly affected oil yield (*P* < 0.01), with the highest values obtained under early to mid-March sowing and the lowest under late April sowing. Seed rate also significantly influenced oil yield, with lower seed rates producing higher oil yield. A significant sowing date × seed rate interaction was observed (Fig. 4A).

### Regression analysis

Multiple linear regression analysis was performed to evaluate the combined effects of climatic variables and agronomic factors on seed yield. The model included mean temperature (Tave), cumulative precipitation during the reproductive phase, and seed rate as predictor variables.

The resulting regression equation was:

\begin{eqnarray*}\text{Seed Yield}=5992.91-220.67\times \mathrm{Tave}+2.19\times \mathrm{Precipitation}-6.13\times \text{Seed Rate}. \end{eqnarray*}

The model indicated that mean temperature had a strong and significant negative effect on seed yield (*P* < 0.001), while precipitation exerted a positive and significant influence (*P* < 0.01). Seed rate also had a significant negative effect on yield (*P* < 0.01), suggesting increased intra-specific competition at higher seed rates.

Model statistics indicated a moderate to strong explanatory power (*R*^2^ = 0.66, *F* = 87.6, *P* < 0.001). Standardized coefficients further revealed that mean temperature was the dominant factor controlling yield variability, followed by precipitation and seed rate. The low variance inflation factor (VIF < 2) values confirmed the absence of multicollinearity among the predictor variables (Table 7).

**Table 7:** Multiple linear regression analysis of seed yield as influenced by climatic variables and seed rate.

Variable	Coefficient (*β*)	Standard error	*t*-value	*P*-value
Intercept	5,992.91	581.63	10.30	<0.001
Mean temperature (°C)	−220.67	23.13	−9.54	<0.001
Total rainfall (mm)	+2.19	0.61	3.58	<0.01
Seed rate (kg ha^−1^)	−6.13	2.01	−3.06	<0.01

Principal component analysis (PCA) was conducted to explore the relationships among mean temperature, precipitation, seed rate, and seed yield. The first two principal components (PC1 and PC2) explained 57.0% and 25.4% of the total variance, respectively, accounting for 82.4% of the overall variability (Fig. 4B). The PCA biplot revealed a clear structure in the data, with seed yield closely aligned with precipitation along the positive direction of PC1, indicating a strong positive association. In contrast, mean temperature was oriented in the opposite direction, suggesting a negative relationship with yield. Seed rate was primarily associated with PC2, suggesting a relatively independent contribution to the overall variation. Overall, the PCA results confirmed the contrasting roles of temperature and precipitation in yield formation, with temperature acting as a limiting factor and precipitation contributing positively under rainfed conditions. The PCA results were consistent with the regression analysis, reinforcing the dominant role of temperature as a limiting factor and the supportive role of precipitation in determining yield variability.

## Discussion

Under semi-arid conditions, temperature appeared to exert a stronger influence on black cumin yield than rainfall. Although rainfall during the late sowing period in 2022 was higher than in 2021, both seed yield and precipitation use efficiency (PUE) tended to decline, suggesting that additional water availability was insufficient to offset the effects of elevated temperatures during the reproductive stage. In line with this pattern, heat use efficiency (HUE) decreased markedly with delayed sowing, indicating reduced efficiency in converting accumulated thermal units into yield. Later sowing likely accelerated thermal accumulation, which shortened the seed filling period and limited yield formation. Elevated temperatures during flowering and seed development may also have increased evapotranspiration rates and reduced water use efficiency, thereby diminishing the contribution of rainfall to yield. Similar reductions in productivity under environmental stress have also been reported in black cumin, where water deficit reduced seed and oil yield while altering physiological and biochemical responses (Bayati et al., 2022; Shahbazi et al., 2022). From a physiological perspective, high temperatures during the reproductive phase can impair pollen viability, reduce fertilization success, and accelerate senescence, ultimately limiting assimilate availability for seed filling (Hasanuzzaman, Nahar & Fujita, 2013; Wahid et al., 2007).

Furthermore, accelerated phenological development under high thermal conditions shortens the duration of key growth stages, further constraining yield potential. These results are in agreement with recent climate assessments indicating that increasing temperature is a primary limiting factor for crop productivity under climate change scenarios (Pörtner et al., 2022). Therefore, under semi-arid environments, optimizing sowing date is essential to avoid exposure of sensitive reproductive stages to terminal heat stress, rather than relying solely on rainfall variability. These findings emphasize that climate adaptive sowing strategies are essential to mitigate the adverse effects of rising temperatures in semi-arid cropping systems. Comparable observations have also been reported in studies conducted under different agroecological conditions in Türkiye. Beyzi & Karer (2020) reported that sowing time significantly affected seed yield and oil content, while Özdemir & Yenikalaycı (2026) found that early spring sowing resulted in the highest seed and oil yields under Eastern Anatolian conditions. Although the optimum sowing period varies among agroecological regions because of differences in climate, these studies consistently demonstrate that sowing within the locally optimal sowing window is essential for avoiding unfavorable environmental conditions during reproductive development and maximizing crop productivity.

The results indicate that phenological development of black cumin is highly sensitive to sowing date, primarily through its interaction with thermal conditions. The reduction in days to flowering under delayed sowing reflects accelerated phenological development due to increased temperature exposure early in the growth cycle. Recent studies on black cumin have likewise demonstrated that environmental stress can accelerate developmental transitions and modify crop performance depending on genotype and growing conditions (Bayati et al., 2022). The shortening of the total growth cycle under late sowing suggests that elevated temperatures accelerate both vegetative and reproductive transitions. This response is consistent with thermal time theory, where increased temperature reduces the duration required to reach developmental thresholds. Comparable responses have also been reported in black cumin by Kizil et al. (2008), who demonstrated that sowing period markedly influenced vegetative growth and yield formation. Their study showed that winter sowing provided a longer vegetative growth period and resulted in higher seed and oil yields than spring sowing. Although the optimum sowing season differed because of contrasting agroecological conditions, the underlying mechanism was similar, with favorable thermal conditions extending the effective growth period and ultimately enhancing reproductive development, seed yield, and oil yield.

Plant height and branching responses further support the dominant role of sowing date. Reduced plant height and lower branching under delayed sowing indicate that shortened vegetative growth limits canopy development and structural biomass accumulation. Similar findings have been reported under heat stress conditions, where rapid thermal accumulation restricts vegetative expansion (Hasanuzzaman, Nahar & Fujita, 2013; Ullah et al., 2024). Likewise, Acar & Karan (2025) reported that improvements in plant height, branch number, capsule number, and thousand-seed weight were closely associated with higher seed yield in black cumin, supporting the importance of vigorous vegetative growth for maximizing reproductive performance. The interaction between sowing date and seed rate suggests that plant density effects are strongly environment dependent. Under early sowing conditions, higher seed rate may promote elongation due to competition for light (Villalobos, Sadras & Fereres, 2017). However, under late sowing, the absence of a strong density response indicates that abiotic stress factors override competition effects.

Branching and capsule formation were strongly reduced under delayed sowing, highlighting the importance of vegetative growth duration for reproductive capacity. These findings suggest that the number of reproductive sites is largely determined by the length of the vegetative phase. Similar patterns have been observed in aromatic and medicinal crops, where optimal sowing windows maximize branching potential and reproductive development (Giridhar et al., 2017; Rahnavard, Sadeghi & Ashrafi, 2010; Tuncturk, Ekin & Turkozu, 2005). Comparable responses were also reported by Toncer & Kizil (2004), who observed that greater branch number and capsule production contributed directly to higher seed yield in black cumin under semi-arid conditions, highlighting the importance of maintaining adequate vegetative growth for reproductive development.

The reduction in the number of seeds per capsule under delayed sowing suggests that reproductive success is sensitive to environmental conditions during flowering. Elevated temperatures at this stage can adversely affect pollen viability and fertilization processes, which may lead to a lower seed set (Hasanuzzaman, Nahar & Fujita, 2013; Ullah et al., 2024). In a similar way, the decrease in thousand seed weight points to possible limitations in assimilate accumulation during the grain-filling period. Under typical conditions, a reduction in seed number per capsule would be expected to increase the amount of assimilates available per seed, potentially resulting in heavier seeds. In the present case, however, higher temperatures may have accelerated senescence while also reducing photosynthetic activity, thereby constraining both assimilate production and translocation. These observations are in line with earlier reports showing that heat stress shortens the seed filling period and reduces final seed mass in oilseed crops (Farooq et al., 2017; Wahid et al., 2007). In addition to sowing date, seed rate also influenced yield formation through intraspecific competition. Higher seed rates reduced both seed number and seed weight, suggesting that increased competition for water and nutrients limited assimilate availability at the plant level. Comparable effects of seed rate have been reported in black cumin. Similarly, Kılıç & Arabacı (2016) demonstrated that both seed rate and sowing date significantly affected seed yield, oil yield, and several yield components, indicating that optimum plant density is essential for balancing interplant competition and resource use efficiency. Mengistu et al. (2021) observed significant effects of seed rate on branching, yield components, and final seed yield, emphasizing the importance of optimizing plant population under rainfed conditions. Similar responses were also reported by Roussis et al. (2022), who found that increasing plant density reduced seed yield and nitrogen use efficiency in *Nigella sativa* grown under Mediterranean conditions. In contrast, lower seed rates appeared to improve resource use efficiency, allowing a greater proportion of assimilates to be allocated to reproductive structures. Similar relationships between seed rate and yield components have been reported in black cumin and other crops grown under semi-arid conditions (Bayati, Karimmojeni & Razmjoo, 2020; Ozer et al., 2020; Tuncturk, Ekin & Turkozu, 2005). The significant interaction between sowing date and seed rate demonstrates that optimal plant density depends on environmental conditions. Under favorable early sowing, reduced competition enhances yield potential, whereas under late sowing, severe abiotic stress limits the benefits of density management. This indicates that environmental constraints may override agronomic adjustments when stress levels exceed critical thresholds.

The results indicate that oil accumulation in black cumin is strongly influenced by sowing date and seed rate, primarily through their effects on environmental conditions and resource availability. The reduction in oil content under delayed sowing suggests that lipid biosynthesis is highly sensitive to thermal stress during the reproductive phase. Comparable responses have also been reported under different agroecological conditions in Türkiye. Kılıç & Arabacı (2016) found that both sowing date and seed rate significantly affected oil content and oil yield, while Kizil et al. (2008) demonstrated that sowing period influenced both fixed and essential oil contents in black cumin. Collectively, these studies support the conclusion that oil accumulation in black cumin is highly responsive to the environmental conditions prevailing during reproductive development.

The observed reduction in oil content can be explained by the adverse effects of high temperatures during seed development, which are known to disrupt enzymatic processes involved in fatty acid synthesis, thereby limiting oil accumulation (Farooq et al., 2017; Hasanuzzaman, Nahar & Fujita, 2013; Wahid et al., 2007). In addition, the shortening of the grain-filling period under late sowing further constrains the duration available for lipid deposition. Similar reductions in oil content under heat and drought stress have been reported in black cumin and other oilseed crops (Abdou et al., 2023; Bayati et al., 2022; Ozer et al., 2020; Shahbazi et al., 2022).

Seed rate effects on oil content highlight the importance of intraspecific competition in determining metabolic allocation. Higher oil content at lower seed rates indicates that improved resource availability per plant enhances energy intensive processes such as lipid biosynthesis. Conversely, increased plant density intensifies competition for water and nutrients, reducing the capacity for secondary metabolite accumulation. Comparable findings have been reported in black cumin and other aromatic crops (Bayati et al., 2022; Rana, 2012; Tuncturk, Ekin & Turkozu, 2005).

Oil yield, as a function of both seed yield and oil content, was primarily driven by sowing date. The strong decline in oil productivity under late sowing reflects the combined negative effects of reduced seed set, lower seed weight, and impaired lipid biosynthesis. These results confirm that sowing time significantly influenced oil yield and seed phytochemical composition in Nigella sativa, with delayed sowing generally reducing oil productivity and optimizing sowing date is critical for maximizing both yield and quality under semi-arid conditions (Rahimi, Zarei & Arminian, 2011; Thani et al., 2024; Toncer & Kizil, 2004).

The multiple regression analysis demonstrated that both climatic conditions and agronomic management significantly influenced yield variability in black cumin. The model showed a moderate to strong explanatory power, indicating that a substantial proportion of yield variation can be attributed to environmental and management factors during the growing period.

Mean temperature (Tave) during the reproductive phase was identified as the dominant factor limiting seed yield, with a strong and highly significant negative effect (b = −220.67, *P* < 0.001). This finding confirms that thermal stress is a primary constraint on crop productivity under semi-arid conditions. Elevated temperatures accelerate phenological development, shorten the seed filling period, and reduce assimilate accumulation, ultimately leading to yield reductions (Farooq et al., 2017; Hasanuzzaman, Nahar & Fujita, 2013; Wahid et al., 2007).

Precipitation, on the other hand, had a positive and significant effect on yield (*b* = 2.19, *P* < 0.01), highlighting the importance of water availability under rainfed conditions. However, its influence was weaker compared to that of temperature, suggesting that increased rainfall alone cannot fully compensate for the negative effects of elevated thermal conditions.

Interestingly, seed rate was also found to significantly affect yield, with a negative co-efficient (b = −6.13, *P* < 0.01). This indicates that higher plant density intensifies intra specific competition for limited resources such as water, nutrients, and light. The interaction between high temperature and increased plant density likely exacerbates stress conditions, particularly under late sowing scenarios, further suppressing yield potential.

Overall, the results indicate that temperature acts as the primary limiting factor, while precipitation plays a supportive role in yield formation. These findings highlight that the interaction between thermal stress and plant density is a key determinant of productivity under semi-arid environments. Therefore, optimizing both sowing date and seed rate is essential to synchronize critical growth stages with favorable climatic conditions and to minimize yield losses under increasing climate variability. Collectively, these results indicate that optimizing sowing date and seed rate provides an effective management strategy to reduce the negative impacts of increasing thermal stress under rainfed semi-arid conditions, thereby improving the resilience of black cumin production systems.

## Conclusions

The results suggest that sowing date plays a major role in determining agronomic performance and oil productivity in black cumin under rainfed semi-arid conditions. Early sowing between early and mid-March tended to extend both vegetative and reproductive phases, allowing the crop to partially avoid exposure to terminal heat stress and unfavorable moisture conditions. This temporal alignment was associated with improved branching, capsule formation, and seed filling, resulting in higher yields compared with delayed sowing dates.

Regression analysis further indicated that mean temperature during the reproductive phase was a key climatic factor limiting yield, whereas precipitation appeared to have a secondary but supportive role. In addition, seed rate influenced productivity, with lower seed rate (10 kg ha^−1^) generally performing better than higher densities, likely due to reduced intra specific competition and more efficient use of available resources.

Taken together, the combined effects of climatic conditions and agronomic practices suggest that yield formation is largely shaped by thermal stress, which may be partially alleviated through appropriate management strategies. Earlier sowing reduces exposure to high temperatures during critical growth stages, while lower seed rates can enhance individual plant performance by improving assimilate allocation. Despite interannual climatic variability, the similar responses observed across the two experimental years suggest a relatively consistent pattern under the studied conditions. Under semi-arid conditions, early sowing combined with lower seed rates may therefore provide a practical approach to improving yield and oil productivity. This combination not only supports key yield components but may also contribute to greater resilience under increasing climate variability.

Overall, these findings provide practical guidance for optimizing black cumin production under rainfed semi-arid environments and may contribute to the development of climate-adaptive management strategies.

### Artificial intelligence (AI) statement

The author declares that ChatGPT (OpenAI) was used solely to improve the language and grammar of the manuscript. The tool was not used for study design, data collection, data analysis, interpretation of the results, or the generation of scientific content. The author carefully reviewed and edited all AI-assisted text and takes full responsibility for the content of the manuscript.

## Supplemental Information

10.7717/peerj.21652/supp-1 Supplemental Information 1 Raw data of black cumin yield, phenology, and oil content under different sowing dates and seed rates Raw experimental observations for black cumin (*Nigella sativa* L.) over two growing seasons (2021–2022). It includes growthand yield parameters (flowering and maturity days,plant height,lateral brunch, number of capsule and seed, seed yield, oil content, and oil yield) across five sowing dates and three seed rates.

10.7717/peerj.21652/supp-2 Supplemental Information 2 Codebook

10.7717/peerj.21652/supp-3 Supplemental Information 3 Daily climate date for research area

## Additional Information and Declarations

### Data Availability

The following information was supplied regarding data availability: The raw measurements are available in the Supplemental Files.
